# Awareness, behavior, and determinants of dietary salt intake in adults: results from the National NCD Monitoring Survey, India

**DOI:** 10.1038/s41598-023-42694-x

**Published:** 2023-09-23

**Authors:** Prashant Mathur, Vaitheeswaran Kulothungan, Anita Nath, K. S. Vinay Urs, Lakshmy Ramakrishnan

**Affiliations:** 1 Indian Council Medical Research–National Centre for Disease Informatics and Research, Nirmal Bhawan, ICMR Complex, Poojanhalli Road, Off NH-7, Adjacent to Trumpet Flyover of BIAL, Kannamangala Post, Bengaluru, 562 110 India; 2 Department of Cardiac Biochemistry, All India Institute of Medical Sciences, New Delhi, India

**Keywords:** Epidemiology, Health care, Medical research, Risk factors

## Abstract

A diet high in sodium contributes to a significant proportion of Disability Adjusted Life Years (DALYs) due to cardiovascular diseases. This paper describes the awareness, behaviour and determinants related to dietary salt intake in an adult population of 18–69 years that were assessed as part of the National NCD Monitoring Survey (NNMS) in India. A sub-sample of 3000 adults selected through simple random sampling from 150 nationally representative Primary Sampling Units (PSUs) was included. Data regarding awareness and behaviour related to dietary salt intake were collected. Urinary sodium excretion in spot urine samples was estimated and used to calculate dietary salt intake. The dietary salt intake’s sociodemographic, behavioural and metabolic determinants were also analysed. Less than one-third of the adults of both genders in all age groups in rural and urban areas were aware that daily high salt intake could affect health. The estimated mean daily salt intake was 8.0 g (8.9 g/day for men and 7.1 g/day for women). The salt intake was significantly higher in men [Adjusted OR = 17.66 (5.24–59.46)], rural areas [Adjusted OR = 6.14 (1.83–20.60)], overweight and obese respondents [Adjusted OR = 17.62 (3.17–98.07)]. The perception of the harmful effects of high salt intake and practices to limit salt intake was low in the study population. The mean daily salt intake was higher than the WHO recommendation of up to 5 g daily. The mean dietary salt intake is high in the Indian population, which calls for planning and implementing control of dietary salt consumption measures.

## Introduction

Cardiovascular diseases (CVD) account for an estimated 28.1% of the total deaths in India. In 2016, 1.63 million deaths were attributable to hypertension compared to 0.78 million deaths in 1990^1^. A diet high in sodium could contribute to 70 million Disability Adjusted Life Years (DALYs’) and 3 million deaths globally because of its association with CVDs^2^. A high sodium level in the diet also increases the risk of stroke and cardiac failure severity^3,4^. Besides the harmful cardiovascular effects of salt consumption, it could also be a potential risk factor for gastric cancer^5,6^. Restricting daily salt intake to the World Health Organization (WHO) recommended level of up to 5 g for adults is a beneficial and cost-saving way to achieve a 25% reduction in the prevalence of raised blood pressure and a 30% reduction in mean population salt intake by 2025. India’s National NCD Monitoring Framework has defined a similar percentage reduction, adapted from WHO’s voluntary global NCD targets^7,8^.

The WHO urges its Member States to strengthen dietary salt reduction strategies^9^. The benefits of attaining the WHO’s objective of reducing daily salt consumption to 5 g per adult by 2030 are substantial. This achievement is estimated to prevent an additional 87,870 premature ischemic heart disease (IHD) cases by 2050 and avoid 126,010 premature strokes^10^. Realising this target would generate an estimated £1260 million in extra healthcare savings alongside these health improvements. While the rationale for salt reduction provided by scientific evidence is robust, the quantum of available data is insufficient to translate such scientific insights into policies for reducing population salt intake. Preventing dietary salt intake must be driven by perceptions, behaviours, and practices influenced by diverse socio-economic and socio-cultural factors. Studies on the assessment of salt intake in India have been done in limited geographical settings; hence the result findings may not apply at a national level^11–13^. The lack of comprehensive data draws attention to the need for a national-level monitoring system to generate evidence on dietary salt intake, knowledge, attitude and practices. Feasible and standard methods of salt estimation incorporated into surveys helps monitor and plan better. These include 24-h urine measurement and spot urine measurement^14^. Although the 24-h urine collection method is acknowledged as the ‘gold standard’ approach for assessing salt intake, collecting urine over a whole day is laborious, costly, inconvenient, and intricate for the individuals involved. Spot urine has been widely used and validated to estimate dietary sodium^15–18^.

The Indian Council of Medical Research—National Centre for Disease Informatics and Research, Bengaluru undertook a comprehensive National NCD Monitoring Survey (NNMS) to establish a monitoring system that addresses the progress toward achieving the national NCD targets^19^. This study aimed to estimate the mean dietary salt intake and assess the awareness, behaviour and determinants of salt intake in an adult population of 18–69 years.

## Materials and methods

### Survey setting, population and sampling

The National NCD Monitoring Survey (NNMS) 2017–18 was conducted on a sample of 12,000 adults in the 18 to 69 age group to generate national-level key NCD-related indicators identified in the national NCD monitoring framework, the details of which are published elsewhere^20^. Urinary sodium excretion in spot urine samples was estimated in a subsample of 3000 adults, selected through simple random sampling in 150 nationally representative Primary Sampling Units, of which 75 were in urban, and the other 75 were in rural areas. Data was collected regarding awareness of the adverse health effects of high salt intake and the importance of lowering dietary salt intake. ‘Behaviour’ alluded to actions regarding salt intake in the daily diet. The sociodemographic, behavioural and metabolic determinants of dietary salt intake estimated from spot urinary samples were also analysed.

### Data and sample collection

The study instrument for the survey was developed from reliable resources related to NCD risk factor surveillance^21–23^. Study participants were recruited after written and verbal consent. Qualified and trained medical social workers collected details at the household, individual and health facilities. Data on behaviours, perceptions and attitudes related to salt use were collected through face-to-face interviews. Camps were organised to conduct anthropometric, blood pressure and blood glucose measurements and collection of urine samples*.* A day before the camp, consenting participants were given 30 mL wide-mouthed labelled sterile urine collection bottles with a barcode specific to a selected adult to avoid sample mismatch. The spot urine sample was gently shaken to ensure homogeneity and transferred to 2 mL collection vials using Pasteur’s pipettes. The vials were then placed in a 9 × 9 storage box in a vaccine carrier containing ice packs for safe transportation at 4 °C to pre-appointed storage facilities, stored at − 20 °C till further shipment to a reference laboratory at the Department of Cardiac Biochemistry, All India Institute of Medical Sciences, New Delhi. The samples were tested in batches of 80 to 100 each.

### Estimation of urinary sodium

The indirect Ion-Selective Electrode (ISE) method was used to estimate urinary sodium (Na) and potassium (K) levels using automated analysers (AU680 Chemistry analyser, Beckman Coulter, CA, USA)^24^. The ISE method has several advantages: it is faster, more accurate, and has lesser sample requirements. The measurable range is 20–200 mmol/L with a precision of < 0.1%. Internal Quality Control (IQC) samples in normal and pathological ranges were run for each batch of urine samples. Jaffe’s method was employed for urinary creatinine level estimation using *Roche analyser (P800 Modular Analytics, Roche Diagnostics, Mannheim, Germany)* through a commercial kit *(Ref. 11,875,418–216, Roche diagnostics, Germany)*. The dietary salt intake from the spot urine sample was estimated using the INTERSALT (International Cooperative Society on Salt and Blood Pressure) equation with potassium for each sex^25^. The value obtained in mmol/L was multiplied by 2.54 to derive the daily mean salt intake in grams per day.

### Data analysis

Data were cleaned using the *IBM Statistical Package for Social Sciences (SPSS) windows—version 22.0.* The statistical analysis using weighted data was carried out in *STATA 14.1* using a complex survey analysis method^26^. Descriptive analysis was used to present results on the behaviour, perception and practices related to salt intake. The data distribution was checked using the graphical representation and then tested for normality with the Shapiro–Wilk test. Mean weighted salt intake comparison between the subgroups (sociodemographic, rural/urban, behavioural, physiological and metabolic risk factors) was made using the Student’s independent samples t-test and Analysis of Variance (ANOVA) test. Logistic regression analysis was carried out to determine the relationship between independent variables, and those variables with a *p*-value < 0.25 were taken in the multivariate analysis^27^. The final multivariate-adjusted model was fitted by adjusting the variables which included age, gender, area of residence, educational status etc.

### Ethics approval

This study was approved by the Institutional Ethics Committee of the Coordinating Centre, ICMR-NCDIR. Approval no: NCDIR/IEC/2017/4 dated 03 February 2017. Before starting the survey, every implementing agency obtained its ethics approval from its Institutional Ethics Committee. Informed consent was obtained from all the study participants. As a post-research benefit, trained social investigators counselled the study participants to reduce salt consumption. They were also provided with a brochure describing tips for a healthy lifestyle in the local language. Since the research involved human participants, it was conducted per the Declaration of Helsinki.

## Results

10,659 adults aged 18–69 years participated in the survey (response rate of 96.3%). Among the subsample of adults selected for the urinary sodium excretion study, 2643 study participants provided spot urine samples, of which 2266 (85.7%) samples were the final number of samples processed and included for analysis. The remaining 377 either had incomplete interviews or the urine samples were contaminated. A flow diagram that indicates the  inclusion of study participants is shown in the supplementary file.

### Awareness and behaviour of the population on dietary salt intake

The proportion of adults who knew that daily high salt intake could affect health was less than one-third among respondents in all age categories, gender, rural–urban areas and those without education. However, over half of the participants were familiar with the importance of lowering salt intake in the diet. The percentage of adults who used extra salt in food or consumed too many salt-containing products was less than a quarter in all the sub-categories of sociodemographic, behavioural and metabolic factors (Table 1).

**Table 1:** Perceptions and behaviours on dietary salt intake.

Characteristics	N	Aware that daily high salt intake affects health			Aware about the importance of lowering salt in diet			Always or often add extra salt to food right before eating it			Far too much or too much consumption of salt containing products		
		n	%	*p*	n	%	*p*	n	%	*p*	n	%	*p*
Sociodemographic factors													
Age groups													
18–44 years	1610	515	32.00	0.102	1084	67.3	**0.002**	252	15.6	0.271	202	12.5	0.445
45–69 years	656	185	28.1		392	59.7		85	12.9		74	11.2	
18–69 years	2266	700	30.9		1476	65.1		336	14.8		276	12.2	
Sex													
Men	1194	382	32.0	0.492	835	69.9	**0.005**	194	16.3	0.253	156	13.1	0.291
Women	1072	317	29.6		641	59.8		142	13.2		119	11.1	
Area of residence													
Rural	1515	455	30.1	0.684	931	61.4	0.021	216	14.2	0.665	186	12.3	0.924
Urban	751	244	32.5		545	72.6		120	16.0		90	11.9	
Educational status													
No formal education	669	133	19.8	** < 0.0001**	353	52.7	** < 0.0001**	88	13.2	0.414	70	10.4	0.218
Received formal education	1597	567	35.5		1123	70.3		248	15.5		206	12.9	
Behavioural factors													
Physical activity													
Sufficient level	1390	405	29.1	0.232	905	65.1	0.999	226	16.2	0.201	179	12.9	0.419
Insufficient level ^a^	876	295	33.7		571	65.1		110	12.6		97	11.0	
Metabolic factors													
BMI categories													
Underweight (< 18.5 kg/m^2^)	432	105	24.4	** < 0.0001**	278	64.3	**0.004**	76	17.7	0.466	60	14.0	0.178
Normal (18.5–24.9 kg/m^2^)	1231	334	27.1		763	61.9		171	13.9		129	10.5	
Overweight and obesity (≥ 25.0 kg/m^2^)	576	254	44.0		420	72.9		86	14.9		83	14.4	
Raised blood glucose^b^													
No	2060	629	30.5	0.553	1332	64.7	0.213	311	15.1	0.444	255	12.4	0.722
Yes	171	58	33.9		123	72.1		21	12.6		19	11.4	
Raised blood pressure^c^													
No	1640	489	29.8	0.202	1030	62.8	**0.020**	257	15.7	0.187	193	11.8	0.671
Yes	620	207	33.4		440	71.0		77	12.5		80	12.9	
Ten-year CVD risk													
< 30	510	156	30.5	0.529	319	62.5	** <0. 0001**	69	13.5	0.970	60	11.7	0.131
≥ 30% or with existing CVD	94	33	35.2		79	83.7		13	13.3		6	6.7	

^a^Insufficient physical activity in adults was defined as proportion of adults aged 18–69 years who spent < 150 min of moderate-intensity physical activity per week* OR < 75 min of vigorous-intensity physical activity per week* OR an equivalent combination of moderate-and-vigorous intensity physical activity accumulating < 600 MET—minutes** per week.

^b^Raised blood glucose in adults aged 18–69 years with fasting blood glucose value ≥ 126 mg/dl including those on medication for raised blood glucose.

^c^Raised blood pressure in adults aged between 18 and 69 years with a “systolic blood pressure ≥ 140 mmHg and/or diastolic blood pressure ≥ 90 mmHg” including those on medication for raised blood Pressure.

Significant values are in bold.

### Weighted mean salt intake estimates according to the sociodemographic profile of the study population

A salt intake of > 5 g/day was observed across all the strata, as shown in Table 2. The estimated mean daily salt intake was 8.0 g (8.9 g/day for men and 7.1 g/day for women). The daily mean salt intake was significantly higher (*p* < 0.0001) in men (8.9 g), employed (8.6 g), current tobacco users (8.3 g), obese (9.2 g) and those with raised blood pressure (8.5 g). The population characteristics and their weighted mean dietary salt intake estimates in India from spot urinary samples according to age groups (18-44 and 45-69 years) and gender are shown in the Supplementary text.

**Table 2:** Population characteristics of adults aged 18–69 years and their weighted mean dietary salt intake estimates in India from spot urinary samples (n = 2266).

Characteristics	n (weighted)	Mean (95% CI) salt in g/day (weighted)	*p*-value
Sociodemographic factors			
Age groups			
18–44 years	1610	7.9 (7.7–8.2)	**0.021**
45–69 years	656	8.2 (8.0–8.4)	
18–69 years	2266	8.0 (7.8–8.2)	
Sex			
Men*	1194	8.9 (8.7–9.2)	** < 0.0001**
Women	1072	7.1 (6.9–7.2)	
Area of residence			
Rural	1515	7.9 (7.7–8.1)	0.09
Urban	751	8.3 (7.9–8.6)	
Educational status			
No formal education	669	7.7 (7.5–8.0)	**0.0001**
Received formal education**	1597	8.2 (7.9–8.4)	
Highest level of education			
Primary	379	8.1 (7.8–8.4)	0.071
Secondary	742	8.1 (7.8–8.4)	
Higher Secondary	238	8.1 (7.6–8.3)	
Graduation and higher	242	8.7 (8.1–9.2)	
Employment status			
Homemaker	703	7.0 (6.8–7.2)	** < 0.0001**
Employed*	1345	8.6 (8.3–8.8)	
Unemployed/student	216	8.0 (7.6–8.4)	
Behavioural factors			
Current tobacco use^a^			
Non-user	1426	7.9 (7.7–8.1)	< 0.0001
User*	840	8.3 (8.1–8.6)	
Current Alcohol consumption^b^			
No	1888	7.9 (7.7–8.2)	**0.03**
Yes	378	8.5 (8.2–8.8)	
Physical activity^c^			
Sufficient level	1390	8.1 (7.9–8.4)	0.075
Insufficient level	876	7.9 (7.6–8.2)	
Practised yoga			
No	2171	8.0 (7.8–8.2)	0.136
Yes	95	8.4 (7.8–9.0)	
Body mass index			
Underweight (< 18.5 kg/m^2^)	432	6.9 (6.7–7.2)	** < 0.0001**
Normal (18.5–24.9 kg/m^2^)	1232	8.0 (7.8–8.2)	
Overweight and obesity (≥ 25.0 kg/m^2^)*	576	9.2 (8.9–9.4)	
Raised fasting blood glucose^d^			
No	2060	8.0 (7.8–8.2)	0.0048
Yes	171	8.4 (8.0–8.8)	
Raised blood pressure^e^			
No	1640	7.9 (7.7–8.1)	** < 0.0001**
Yes*	619	8.5 (8.2–8.8)	
Reported raised cholesterol			
No	67	8.2 (7.8–8.6)	0.0162
Yes	19	8.9 (8.3–9.5)	
Ten-year Cardiovascular disease risk			
< 30	510	8.4 (8.2–8.7)	
≥ 30% or with existing CVD	94	8.4 (7.8–9.0)	

*p < 0.001.

**p < 0.05.

^a^Current tobacco use:use of any form of tobacco (smoke and/or smokeless) in the last 12 months preceding the survey.

^b^Current alcohol use: consumption of alcohol in the last 12 months preceding the survey.

^c^Insufficient physical activity in adults was defined as proportion of adults aged 18–69 years who spent < 150 min of moderate-intensity physical activity per week* OR < 75 min of vigorous-intensity physical activity per week* OR an equivalent combination of moderate-and-vigorous intensity physical activity accumulating < 600 MET- minutes** per week.

^d^Raised blood glucose in adults aged 18–69 years with fasting blood glucose value ≥ 126 mg/dl including those on medication for raised blood glucose.

^e^Raised blood pressure in adults aged between 18 and 69 years with a “systolic blood pressure ≥ 140 mmHg and/or diastolic blood pressure ≥ 90 mmHg” including those on medication for raised blood Pressure.

Significant values are in bold.

### Association between sociodemographic, behavioural, physiological and metabolic factors with salt intake

A salt intake of > 5 g per day was significantly higher in men than women [Unadjusted OR = 6.69 (2.52–17.72)]; [Adjusted OR = 17.66 (5.24–59.46)], among rural participants than in urban [Adjusted OR = 6.14 (1.83–20.60)], overweight and obese persons [Unadjusted OR = 10.95 (2.24–53.50); Adjusted OR = 17.62 (3.17–98.07)]. The findings are presented in Table 3.

**Table 3:** Association between demographic, behavioural and metabolic risk factors with salt intake (g/day).

Predictors	Bivariate		Multivariate	
	Unadjusted OR (95% CI)	*p*-value	Adjusted OR (95% CI)	*p*-value
Sociodemographic factors				
Age groups				
18–44 years	1		1	
45–69 years	1.57 (0.84–2.95)	0.159	0.57 (0.19–1.67)	0.303
Sex				
Women	1		1	
Men	6.69 (2.52–17.72) **	** < 0.0001**	17.66 (5.24–59.46)**	** < 0.0001**
Area of residence				
Urban	1		1	
Rural	1.61(0.65–4.00)	0.304	6.14 (1.83–20.60) *	**0.004**
Educational status				
Received formal education	1		1	
No formal education	1.02 (0.65–1.61)	0.928	2.46 (0.94–6.44)	0.066
Employment status				
Unemployed	1		1	
Employed	6.10 (2.48–14.97)	0.001	4.45 (1.48–13.38)	0.963
Homemaker	1.27 (0.53–3.05)	0.597	4.34 (1.61–11.73) *	0.004
Behavioural risk factors				
Current tobacco use^a^				
Non-user	1		1	
User	1.68 (0.89–3.19)	0.111	0.78 (0.35–1.73)	0.54
Current alcohol consumption^b^				
No	1		1	
Yes	1.40 (0.51–3.87)	0.511	0.78 (0.20–3.03)	0.719
Physical activity^c^				
Sufficient level	1		1	
Insufficient level	0.60 (0.33–1.08)	0.09	1.42 (0.67–3.00)	0.357
Metabolic risk factors				
BMI categories				
Normal (18.5–24.9 kg/m^2^)	1		1	
Underweight (< 18.5 kg/m^2^)	0.10 (0.04–0.26)**	** < 0.0001**	0.07 (0.03–0.21)**	** < 0.0001**
Overweight and Obesity (≥ 25.0 kg/m^2^)”	10.95 (2.24–53.50)**	** < 0.0001**	17.62 (3.17–98.07)**	** < 0.0001**
Raised blood glucose^d^				
No	1		1	
Yes	1.64 (0.55–4.93)	0.376	2.38 (0.31–18.17)	0.402
Raised blood pressure^e^				
No	1		1	
Yes	1.25 (0.63–2.45)	0.525	0.61 (0.22–1.72)	0.347
Ten-year Cardiovascular disease risk				
> 30	1			
≥ 30% or with existing CVD	0.52 (0.10–2.67)	0.426		

*BMI* Body mass index; *CVD* Cardio vascular disease; *OR* Odds ratio

*p < 0.001, **p < 0.05.

^a^Current tobacco use: use of any form of tobacco (smoke and/or smokeless) in the last 12 months preceding the survey.

^b^Current alcohol use: consumption of alcohol in the last 12 months preceding the survey.

^c^Insufficient physical activity in adults was defined as proportion of adults aged 18–69 years who spent < 150 min of moderate-intensity physical activity per week* OR < 75 min of vigorous-intensity physical activity per week* OR an equivalent combination of moderate-and-vigorous intensity physical activity accumulating < 600 MET- minutes** per week.

^d^Raised blood glucose in adults aged 18–69 years with fasting blood glucose value ≥ 126 mg/dl including those on medication for raised blood glucose.

^e^Raised blood pressure in adults aged between 18 and 69 years with a “systolic blood pressure ≥ 140 mmHg and/or diastolic blood pressure ≥ 90 mmHg” including those on medication for raised blood Pressure.

Significant values are in bold.

### Consumption of high salt containing foods among adults

**Figure 1:**
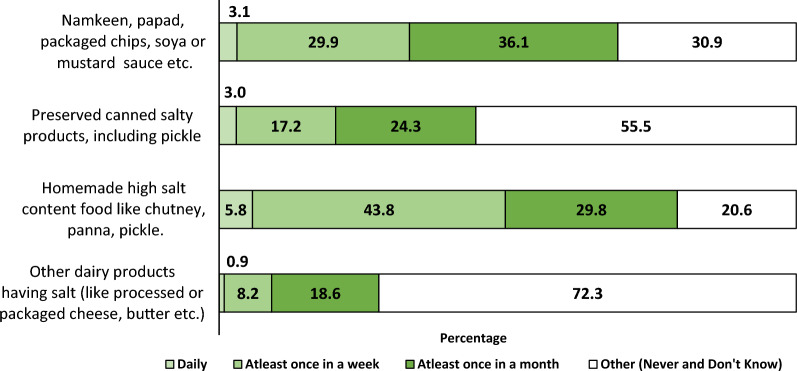
Frequency of consumption of high salt containing foods among adults.

Approximately 43.8% of adults indicated that they consumed high-salt homemade food items every week (1–6 days per week), while 36.1% reported consuming salty snacks like *namkeen*, papad, and packaged chips at least once a month (1–3 days or less than once a month), as seen in Fig. 1.

## Discussion

The present study provides a national-level description of the awareness and behaviour of dietary salt intake and its determinants in terms of the sociodemographic (age, sex, area, education and employment), behavioural (current tobacco and alcohol use, physical activity and yoga practice), physiological (body mass index, blood pressure) and metabolic factors (blood glucose and cholesterol level) in the Indian population. While most respondents knew the importance of reducing dietary salt consumption, the proportion of those who perceived that a daily high salt intake could affect one’s health was low. The pattern of perception and behaviour did not substantively vary according to sociodemographic, behavioural and metabolic factors. These findings are similar to the survey conducted in Delhi, Haryana, and Andhra Pradesh, in which salt intake-related awareness and behaviour did not vary across different levels of education^28^. Studies done in specific and geographically limited settings have shown varying results. Close to two-thirds (64%) of the participants residing in an urban slum in Chandigarh, North India, did not consider lowering the salt intake important^29^. In another community-based study in North India, 46·6% from rural Haryana and 45·1% from urban Delhi were aware of the need to reduce dietary salt content^30^. In our study, it was positive to note that less than a third of the respondents affirmed that they added extra salt to their food or consumed far too many salty items. However, less than half of the study participants practised measures to control dietary salt intake. The most commonly adopted step was avoiding meals outside of the home (Supplementary Table S1).

In the present study, the weighted mean salt intake was more than the WHO-recommended salt intake of up to 5 g per day across all groups categorised according to the sociodemographic, behavioural and metabolic factors groups. Similar findings have been reported in other studies done in India. A systematic review of the mean salt intake in India found the observed value of 11 g per day^31^. Analogous study findings for mean dietary salt intake were reported in Andhra Pradesh (8.72 g/day) and Delhi, Haryana (5.62 g/day), which was calculated from the average estimates from two 24-h dietary recall surveys. The Chennai Urban Rural Epidemiology (CURE) study also observed the mean salt intake as 8.5 g/day, using the food frequency questionnaire (FFQ)^32^. The mean salt intake in women in an urban resettlement colony in Delhi was 7.6 g/day based on spot urine sodium measurements^13^. Some methods, such as dietary surveys, are time-consuming and have a low validity^33,34^. Even samples of dried urine obtained from spot collection samples are a clinically valid and convenient alternative to liquid urine to analyse sodium concentration under field conditions^35^. The salt intake was significantly higher in men, the rural population, the employed, overweight and obese. Similar findings on higher salt intake in males^36,37^, rural people^30^, and obese individuals^38,39^ were seen in other studies.

The salt added during food preparation constitutes the primary source of dietary salt in low- and middle-income such as India^40^. Studies conducted in high-income countries observed the average salt intake of 9–12 g daily; processed foods comprised the primary source^41,42^. However, with the socio-economic progress and changing lifestyle, India is witnessing a ‘nutrition transition’ wherein there is a decrease in the consumption of wholesome foods and an increasing reliance on packaged and processed food^43,44^.

Our study showed that the mean dietary salt intake to be high in the Indian population, which calls for planning and implementing control of dietary salt consumption measures. The results pave the way for the planning and implementing control of dietary salt consumption measures in the Indian population. A universal reduction in the dietary sodium consumption of at least 1.2 g per day would help achieve a 50% reduction in the proportion of persons who require anti-hypertensive treatment^45^. An awareness of the adverse health effects could substantially influence the willingness to curb excessive salt consumption. The findings on perceptions and practices related to salt intake could help design effective consumer education and awareness programmes. However, awareness generation alone may not always successfully bring about behavioural change^46^. A multisectoral and coordinated approach is essential to ensure actual limitations of salt intake. Intensive efforts should be leveraged through National Multi-sectoral Action Plan, which includes salt reduction as an active component. The Food Safety and Standard Authority of India (FSSAI) has played a significant role in generating awareness and encouraging community involvement through ‘The Salt Challenge, Every Pinch Counts’^47^. Instead of sodium chloride, low sodium salt substitutes have potassium chloride for reducing sodium content, although these are more expensive than regular salt. Framing of policies to scale up the availability and affordability of low sodium salt substitutes should be promoted to ascertain a reduction in the mean population salt intake. Other potential measures include mass awareness, training of food vendors and clear labelling of sodium content in packaged foods.

## Strength and limitations

Our study was conducted in a nationally representative sample wherein dietary sodium intake was estimated from spot urine samples, a validated method used to assess dietary sodium intake. The population mean was calculated using sampling weights; thus, the study findings could be generalised at a population level and used to plan and implement dietary salt control measures. The information on awareness and behaviour of salt intake was self-reported and could be subjected to information bias. The study did not capture data on the actual dietary sources of salt in food items and condiments for any correlation analysis.

### Supplementary Information

Supplementary Information.

## Data Availability

The datasets generated during and/or analysed during the current study are available from the corresponding author on reasonable request.
