# Supplementary material for: Awareness, behavior, and determinants of dietary salt intake in adults: results from the National NCD Monitoring Survey, India
Source: Sci Rep. 2023 Sep 23;13:15890. doi: 10.1038/s41598-023-42694-x (PMC10517942; doi:10.1038/s41598-023-42694-x)
Supplement: Supplementary file 1 — Supplementary Information. [file 41598_2023_42694_MOESM1_ESM.docx]

**Supplementary table 1a: Population characteristics (18-44 years & 45-69 years) and their weighted mean dietary salt intake estimates in India from spot urinary samples (n= 2266) by age groups**

| 18-44 years | | | | | 45-69 years | | | |
| --- | --- | --- | --- | --- | --- | --- | --- | --- |
| **Characteristics** | **n (weighted)** |  | **Mean (95% CI) salt in g/day (weighted)** | **p-value** | **n (weighted)** |  | **Mean (95% CI) salt in g/day (weighted)** | **p-value** |
| **Sociodemographic factors** | |  |  |  |  |  |  |  |
| **Sex** |  |  |  |  |  |  |  |  |
| Men | 866 |  | 8.7 (8.4-9.0) | **<0.001** | 328 |  | 9.5 (9.3-9.7) | **<0.001** |
| Women | 743 |  | 7.1 (6.9-7.3) |  | 328 |  | 7.0 (6.8-7.2) |  |
| **Area of residence** |  |  |  |  |  |  |  |  |
| Rural | 1081 |  | 7.8 (7.6-8.1) | **<0.001** | 434 |  | 8.2 (8.0-8.4) | 0.172 |
| Urban | 528 |  | 8.2 (7.8-8.6) |  | 223 |  | 8.4 (8.0-8.8) |  |
| **Educational status** |  |  |  |  |  |  |  |  |
| No education | 320 |  | 7.7 (7.4-8.0) | **0.009** | 350 |  | 7.8 (7.5-8.0) | **<0.001** |
| Received some education | 1290 |  | 8.0 (7.8-8.3) |  | 307 |  | 8.8 (8.6-9.1) |  |
| **Highest level of education** | |  |  |  |  |  |  |  |
| Primary | 271 |  | 7.9 (7.6-8.3) | **<0.001** | 108 |  | 8.5 (8.1-8.8) | **0.006** |
| Secondary | 602 |  | 7.9 (7.6-8.2) |  | 140 |  | 8.9 (8.4-9.3) |  |
| Higher Secondary | 211 |  | 7.8 (7.4-8.2) |  | 27 |  | 9.6 (9.2-10.0) |  |
| Graduation and higher | 212 |  | 8.5 (7.8-9.2) |  | 30 |  | 9.6 (8.9-10.2) |  |
| **Employment status** |  |  |  |  |  |  |  |  |
| Homemaker | 490 |  | 7.1 (6.8-7.3) | **<0.001** | 213 |  | 6.9 (6.7-7.1) | **<0.001** |
| Employed | 959 |  | 8.5 (8.2-8.8) |  | 386 |  | 8.8 (8.6-9.1) |  |
| Unemployed/student | 161 |  | 7.5 (7.1-8.0) |  | 55 |  | 9.2 (8.6-9.9) |  |
| **Behavioural factors** |  |  |  |  |  |  |  |  |
| **Current tobacco use** |  |  |  |  |  |  |  |  |
| Non-user | 1046 |  | 7.8 (7.6-8.0) | **<0.001** | 379 |  | 8.0 (7.8-8.2) | **<0.001** |
| User | 563 |  | 8.2 (7.9-8.6) |  | 277 |  | 8.6 (8.2-8.9) |  |
| **Current Alcohol consumption** | |  |  |  |  |  |  |  |
| No | 1338 |  | 7.9 (7.6-8.1) | **0.005** | 549 |  | 8.1 (7.9-8.3) | **<0.001** |
| Yes | 271 |  | 8.2 (7.9-8.6) |  | 107 |  | 9.2 (8.8-9.6) |  |
| **Physical activity** |  |  |  |  |  |  |  |  |
| Sufficient level | 1014 |  | 8.0 (7.8-8.3) | **0.007** | 376 |  | 8.4 (8.1-8.7) | **0.011** |
| Insufficient level | 595 |  | 7.8 (7.4-8.2) |  | 281 |  | 8.0 (7.7-8.3) |  |
| **Practised Yoga** |  |  |  |  |  |  |  |  |
| No | 1548 |  | 7.9 (7.7-8.2) | **0.021** | 623 |  | 8.3 (8.0-8.5) | 0.879 |
| Yes | 61 |  | 8.5 (7.6-9.4) |  | 33 |  | 8.3 (7.8-8.8) |  |
| **Body Mass Index** |  |  |  |  |  |  |  |  |
| Underweight (< 18.5 Kg/m^2^) | 324 |  | 6.8 (6.5-7.1) | **<0.001** | 108 |  | 7.4 (6.8-7.9) | **<0.001** |
| Normal (18.5 – 24.9 Kg/m^2^) | 877 |  | 7.9 (7.7-8.1) |  | 354 |  | 8.2 (8.0-8.4) |  |
| Overweight and obesity (≥ 25.0 Kg/m^2^) | 384 |  | 9.3 (9.0-9.6) |  | 192 |  | 8.9 (8.5-9.3) |  |
| **Raised fasting blood glucose** | |  |  |  |  |  |  |  |
| No | 1509 |  | 7.9 (7.7-8.2) | **0.008** | 551 |  | 8.3 (8.0-8.5) | 0.654 |
| Yes | 74 |  | 8.5 (7.8-9.2) |  | 96 |  | 8.4 (8.0-8.8) |  |
| **Raised blood pressure** |  |  |  |  |  |  |  |  |
| No | 1279 |  | 7.8 (7.6-8.0) | **<0.001** | 362 |  | 8.1 (7.9-8.3) | **0.019** |
| Yes | 325 |  | 8.6 (8.2-8.9) |  | 295 |  | 8.5 (8.1-8.8) |  |
| **Reported raised cholesterol** | |  |  |  |  |  |  |  |
| No | 1605 |  | 7.9 (7.7-8.2) | 0.634 | 642 |  | 8.2 (8.0-8.4) | 0.114 |
| Yes | 5 |  | 8.4 (7.2-9.5) |  | 14 |  | 9.1 (8.4-9.8) |  |
| **Ten-year Cardiovascular disease risk** | | |  |  |  |  |  |  |
| <30 | 122 |  | 8.9 (8.5-9.3) | 0.095 | 389 |  | 8.3 (8.0-8.6) | 0.422 |
| ≥30% or with existing CVD | 31 |  | 8.2 (6.9-9.6) |  | 64 |  | 8.5 (8.0-9.0) |  |

**Supplementary table 1b: Population characteristics and their weighted mean dietary salt intake estimates in India from spot urinary samples (n= 2266) by gender**

| MALE | | | | | FEMALE | | | |
| --- | --- | --- | --- | --- | --- | --- | --- | --- |
| **Characteristics** | **n (weighted)** |  | **Mean (95% CI) salt in g/day (weighted)** | **p-value** | **n (weighted)** |  | **Mean (95% CI) salt in g/day (weighted)** | **p-value** |
| **Sociodemographic factors** | |  |  |  |  |  |  |  |
| **Age groups** |  |  |  |  |  |  |  |  |
| 18-44 years | 866 |  | 8.7 (8.4-9.0) | **<0.001** | 743 |  | 7.1 (6.9-7.3) | 0.344 |
| 45-69 years | 328 |  | 9.5 (9.3-9.7) |  | 328 |  | 7.0 (6.8-7.2) |  |
| 18-69 years |  |  |  |  |  |  |  |  |
| **Area of residence** |  |  |  |  |  |  |  |  |
| Rural | 793 |  | 8.8 (8.5-9.1) | **0.001** | 722 |  | 7.0 (6.8-7.2) | **0.004** |
| Urban | 401 |  | 9.2 (8.7-9.6) |  | 350 |  | 7.2 (6.9-7.6) |  |
| **Educational status** |  |  |  |  |  |  |  |  |
| No education | 214 |  | 9.1 (8.8-9.5) | 0.106 | 455 |  | 7.1 (6.9-7.3) | 0.606 |
| Received some education | 980 |  | 8.9 (8.6-9.1) |  | 617 |  | 7.0 (6.8-7.2) |  |
| **Highest level of education** | |  |  |  |  |  |  |  |
| Primary | 209 |  | 8.8 (8.4-9.3) | **<0.001** | 170 |  | 7.2 (6.8-7.6) | **0.002** |
| Secondary | 444 |  | 8.7 (8.4-9.1) |  | 298 |  | 7.2 (6.9-7.4) |  |
| Higher Secondary | 158 |  | 8.7 (8.3-9.2) |  | 80 |  | 6.5 (6.1-7.0) |  |
| Graduation and higher | 168 |  | 9.4 (8.7-10.1) |  | 73 |  | 6.8 (6.4-7.3) |  |
| **Employment status** |  |  |  |  |  |  |  |  |
| Homemaker | 7 |  | 9.8 (9.4-10.1) | 0.236 | 695 |  | 7.0 (6.8-7.2) | **<0.001** |
| Employed | 1037 |  | 8.9 (8.7-9.2) |  | 308 |  | 7.3 (7.1-7.6) |  |
| Unemployed/student | 147 |  | 8.7 (8.3-9.2) |  | 69 |  | 6.3 (5.8-6.8) |  |
| **Behavioural factors** |  |  |  |  |  |  |  |  |
| **Current tobacco use** |  |  |  |  |  |  |  |  |
| Non-user | 517 |  | 9.2 (9.0-9.5) | **<0.001** | 908 |  | 7.1 (6.9-7.3) | 0.146 |
| User | 677 |  | 8.7 (8.4-9.0) |  | 163 |  | 6.9 (6.6-7.2) |  |
| **Current Alcohol consumption** | |  |  |  |  |  |  |  |
| No | 845 |  | 9.0 (8.8-9.3) | **0.004** | 1043 |  | 7.1 (6.9-7.2) | 0.072 |
| Yes | 349 |  | 8.7 (8.4-8.9) |  | 29 |  | 6.6 (5.7-7.5) |  |
| **Physical activity** |  |  |  |  |  |  |  |  |
| Sufficient level | 866 |  | 8.8 (8.6-9.1) | **0.001** | 524 |  | 7.1 (6.8-7.3) | 0.889 |
| Insufficient level | 328 |  | 9.2 (8.8-9.6) |  | 548 |  | 7.0 (6.9-7.2) |  |
| **Practised Yoga** |  |  |  |  |  |  |  |  |
| No | 1133 |  | 8.9 (8.7-9.1) | 0.931 | 1039 |  | 7.0 (6.9-7.2) | 0.068 |
| Yes | 61 |  | 8.9 (8.1-9.8) |  | 33 |  | 7.5 (7.2-7.8) |  |
| **Body Mass Index** |  |  |  |  |  |  |  |  |
| Underweight (< 18.5 Kg/m^2^) | 226 |  | 7.5 (7.2-7.9) | **<0.001** | 206 |  | 6.3 (6.0-6.7) | **<0.001** |
| Normal (18.5 – 24.9 Kg/m^2^) | 672 |  | 8.8 (8.6-9.1) |  | 560 |  | 7.0 (6.8-7.1) |  |
| Overweight and obesity (≥ 25.0 Kg/m^2^) | 293 |  | 10.3 (10.1-10.5) |  | 283 |  | 8.0 (7.8-8.2) |  |
| **Raised fasting blood glucose** | |  |  |  |  |  |  |  |
| No | 1079 |  | 8.9 (8.6-9.1) | **0.02** | 981 |  | 7.0 (6.9-7.2) | 0.216 |
| Yes | 95 |  | 9.4 (8.9-9.8) |  | 76 |  | 7.3 (6.9-7.6) |  |
| **Raised blood pressure** |  |  |  |  |  |  |  |  |
| No | 842 |  | 8.7 (8.4-9.0) | **<0.001** | 798 |  | 7.0 (6.8-7.2) | **0.008** |
| Yes | 348 |  | 9.5 (9.2-9.8) |  | 272 |  | 7.2 (7.0-7.5) |  |
| **Reported raised cholesterol** | |  |  |  |  |  |  |  |
| No | 1182 |  | 8.9 (8.7-9.1) | **0.11** | 1065 |  | 7.1 (6.9-7.2) | 0.815 |
| Yes | 12 |  | 9.8 (9.2-10.4) |  | 6 |  | 7.2 (6.4-8.0) |  |
| **Ten-year Cardiovascular disease risk** | | |  |  |  |  |  |  |
| <30 | 266 |  | 9.6 (9.4-9.9) | **0.092** | 244 |  | 7.1 (6.9-7.3) | **0.229** |
| ≥30% or with existing CVD | 55 |  | 9.2 (8.3-10.0) |  | 39 |  | 7.4 (6.7-8.0) |  |


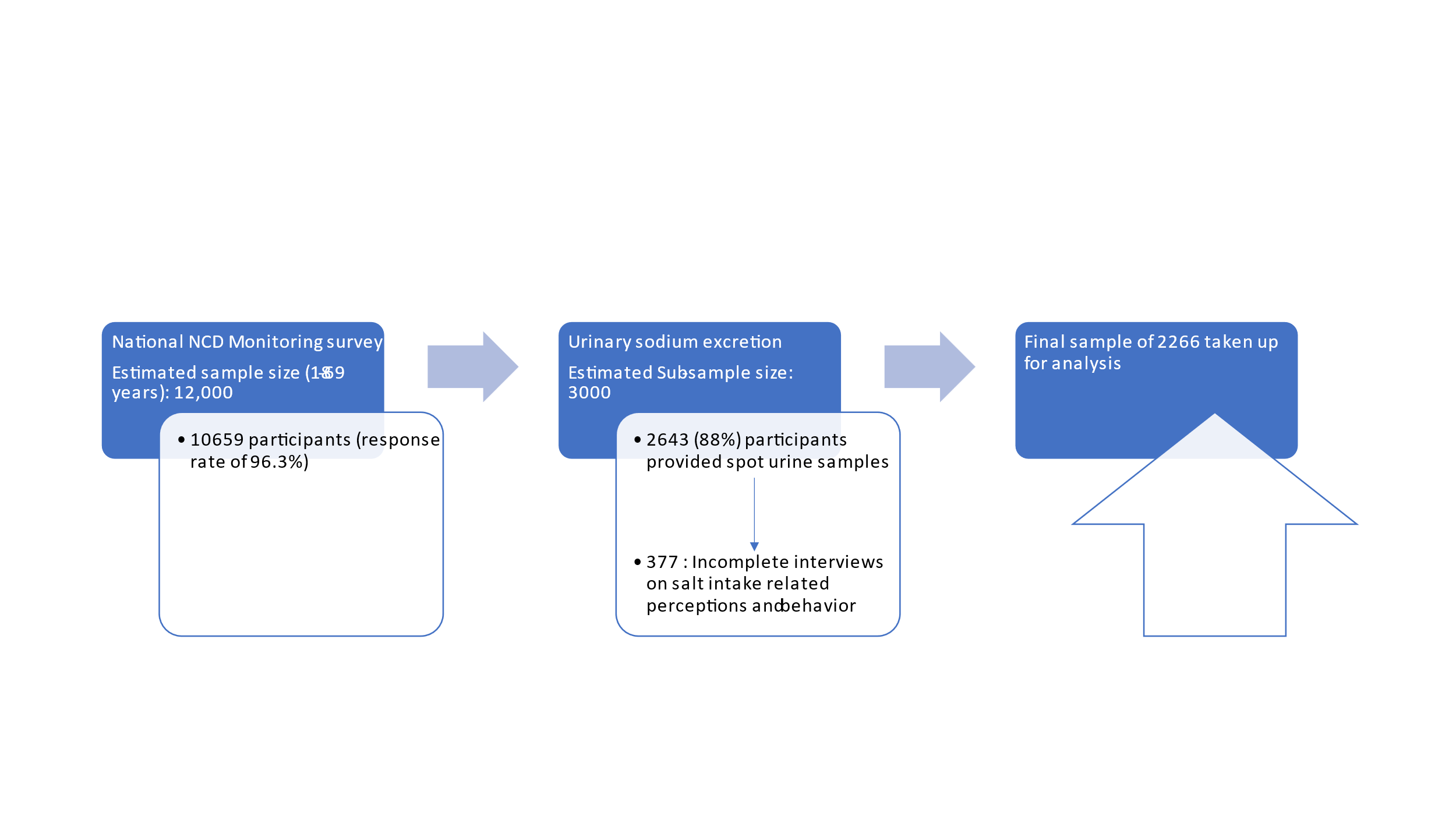


**Figure : Flow diagram on inclusion of study participants**
